# Are epidermal stem cells unique with respect to aging?

**DOI:** 10.18632/aging.100082

**Published:** 2009-08-19

**Authors:** Doina Racila, Jackie R. Bickenbach

**Affiliations:** Department of Anatomy and Cell Biology, the University of Iowa, Iowa City, IA 52242, USA

**Keywords:** epidermis, ROS, keratinocyte

## Abstract

Epidermal stem cells are a population of somatic stem cells responsible for maintaining and repairing the epidermis of the skin. A malfunctioning epidermal stem cell compartment results in loss of the epidermis and death of the whole organism. Since the epidermis continually renews itself by sloughing a layer of cells every day, it is in a constant state of cellular turnover and requires continual cell replacement for life. Thus, maintaining a pristine epidermal stem cell population is of prime importance, even during aging. Unlike stem cells from internal tissues, epidermal stem cells show little response to aging. They do not appear to decrease in number or functionality with age, and do not show changes in gene expression, developmental responsiveness, or age-associated increases of reactive oxygen species. Thus, epidermal stem cells may be a unique somatic stem cell.

## Why epidermal stem cells might be unique

How an organism and its cells age is an ongoing debate. One view is that organisms age because their cells accumulate a series of accidental, but detrimental events throughout life. Another view espouses that cells follow an established program of genetic and epigenetic changes which slowly, but deliberately result in loss of cellular repair mechanisms and ultimately in organismal death (for review see [1]). A further point to consider is do organisms proceed through the aging process because they gradually lose their stem cells as they age or because their stem cells gradually change their function? Evidence from intestinal and hematopoietic stem cells suggest that both may happen: the number of stem cells decreases and their function changes with increasing age [2-5]. However, work from our lab and others suggests that this is not the case for all somatic stem cells, that mammalian epidermal stem cells appear to resist the aging process. These adult stem cells show no loss in numbers, no changes in gene expression or cellular function, and no changes in telomere length with respect to age [6-8].

Perhaps epidermal stem cells are unique among somatic stem cells because no matter how old the skin is, the epidermis must continually replace itself with correctly functioning cells in order to protect the organism from the outside world [9]. Mammalian epidermis sloughs around one layer of cells every day. The sloughed cells are replaced through proliferation of cells in the lower layers [10]. If epidermal cell replacement ceases for any length of time, the mammal will die. This continual need for replacement cells is met by epidermal stem cell proliferation followed by a series of amplifying cell divisions of the daughter cells. Because the epidermis lives a long time, epidermal stem cells are by default very long lived. In fact, they can essentially "outlive" the mammal from which they came, as evidenced by old skin grafted to young individuals living past the death of the donor  [11,12]. Having epidermal stem cells resist aging may be an epidermal protective mechanism evolved against unexpected extension of life.

## Epidermal stem cells remain undifferentiated and functional into old age

We have known for nearly thirty years that stratified squamous epithelia contain slowly or intermittently cycling keratinocytes. These cells are identified as label-retaining cells (LRCs) by the long term retention of a tritiated thymidine or bromodeoxyuridine (BrdU) label [13-15]. Morphologically, LRCs look like undifferentiated primitive cells with a large nuclear to cytoplasmic ratio and have the characteristics of epidermal stem cells [15,16]. Furthermore, in neonatal epithelia the somatic epidermal stem cells are morphologically the same as those in adult epithelia [17]. It is thought that a somatic stem cell asymmetrically divides producing one daughter identical to itself and one daughter cell that increases its proliferative rate to maintain the tissue. Although there is no direct evidence for this phenomenon in the epidermis, evidence does exist in the small intestinal epithelium [18]. Asymmetric cell division was predicted more than three decades ago as an intrinsic way for stem cells in continuously renewing tissues, such as the epidermis, to protect their DNA by minimizing DNA replication related defects [19]. This may explain why stem cells from both neonatal and adult epidermis rarely enter the cell cycle even though the epidermis requires continual cell replacement. Ninety-six percent of the epidermal stem cells from both age groups remain in G1 of the cell cycle, whereas only 4% of the cells are cycling in S-G2/M at any given time [17]. Instead it is the transit amplifying daughter population in both age groups that is highly proliferative, with 15% of these cells in S-G2/M [17]. Recent in vivo studies confirm that the epidermal stem cell does not change its repopulating characteristics with age; instead it is the aging transit amplifying daughter cell that changes its kinetics [20].

Our method preferentially selects for the LRC population, and thus highly enriches for the epidermal stem cells [16]. It combines and refines two previous published methods  The first method showed that the long term repopulating hematopoietic stem cells were the cells that excluded the vital dye Hoechst 33342 via the ABCG2 transporter [21,22]. The second method showed that the smallest epidermal keratinocytes were the cells that produced the largest clonogenic potential in vitro [23]. Epidermal cells, isolated by combining these two methods, not only recapitulate a functional epidermis, but also show multipotency when injected into a developing mouse blastocyst [6,16]. This is irrespective of the cell's age. Analysis shows that gene expression does not change between young adult and old adult mouse epidermal cells [7]. These combined findings suggest that epidermal stem cells maintain their functionality well into old age.  Our results differ from recent reports of hematopoietic stem and progenitor cells in which gene expression not only changed with age in both populations, but was directly associated with replicative senescence [5]. Such findings emphasize the difference between epidermal stem cells and other somatic stem cells, especially with respect to aging.

## Epidermal stem cells remain developmentally responsive irrespective of their age

We have shown that epidermal stem cells isolated from newborn or aging mouse skin have a similar plasticity response when injected in a developing blastocyst environment  [6,7]. The labeled cells are found incorporated into tissues from all three germ layers. The injected stem cells alter their epidermal profile and express proteins of the tissues into which they develop in vivo. Furthermore, the cells or their progeny are retained for the life of the resultant mouse. Thus, something in the developmental environment of the blastocyst is able to reprogram the injected epidermal cells. This phenomenon is unique to the epidermal stem cells as none of the other basal keratinocytes or their progeny are found in any of the adult mouse tissues.

It was not determined in these experiments whether it is contact with the cells in the blastocyst or a response to something secreted by the cells that reprogram the epidermal cells. In vitro, exposure to cell-free extracts or conditioned media from pluripotent cells results in reprogramming of differentiated cells [24,25]. The treated cells increase their developmental potency and can be specifically directed to differentiate into neuronal cells or B lymphocytes in vitro [25,26]. In the B lymphocyte experiment, the epidermal stem cells were shown to permanently change their genome by deleting VDJ segments from the heavy chain immunoglobulin locus. Only the epidermal stem cells respond to these stimuli, and their age is irrelevant to their response.  These findings indicate two things: first, that epidermal stem cells have retained a remarkable developmental potency and second, this ability to transform into other cell types in response to environmental stimuli is not lost with age.

How epidermal stem cells maintain the ability to be developmentally responsive into old age is not understood. One potential mechanism might be to control the levels of reactive oxygen species within the cellular borders. We have three reasons for posing this possible scenario. First, oxidative stress has been associated with increased aging at the molecular level as shown by the deletion of the superoxide dismutase 1 (Sod1) gene producing a decrease in the lifespan of mice [27]. Superoxide dismutase 1 is an enzyme required to catalyze the dismutation of superoxide, a reactive oxygen species (ROS). Second, it is believed that changes in levels of oxygen can change the developmental potential of a cell via new epigenetic programming (reviewed in [28]). Third, although we found no differences in gene expression between young and old epidermal stem cells, we did find that these cells had much higher expression of Sod1 than did the other basal keratinocytes [7].

**Figure 1.**
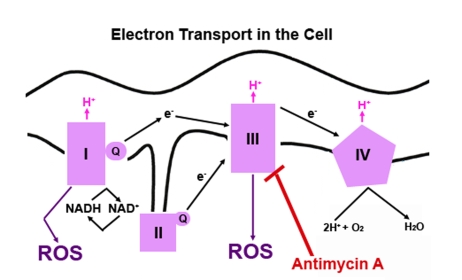
**A simple diagram depicting electron transport in mammalian cells. ** The redox potential increases as the electrons move through each complex located in the inner membrane of the mitochondria. NADH, an electron donor, donates two electrons. The electrons flow through the four complexes causing hydrogen (H^+^) to be pumped across the inner mitochondrial membrane to favor free energy. At each step, the free electron can be picked up by oxygen (O_2_), which will convert O_2_ to superoxide, a highly reactive oxygen species (ROS). Electron transfer can be blocked at the complexes by several different compounds. Antimycin A blocks transfer of electrons at complex III.

**Figure 2.**
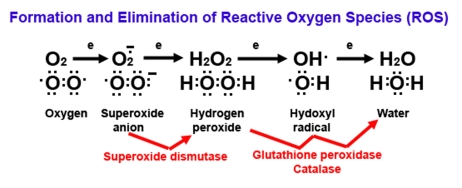
**Diagram of reactive oxygen (ROS) formation.** Oxygen (O_2_) plays a major role in the formation of ROS because O_2_ has unpaired electrons (represented by single dots). When O_2_ picks up an electron, it becomes superoxide, an extremely reactive anion. Superoxide dismutase catalyzes the dismutation reaction of superoxide to hydrogen peroxide, which is further catalyzed to the highly reactive hydroxyl radical and ultimately to water by glutathione peroxidase and catalase enzymes. Superoxide, hydrogen peroxide, and hydroxyl radicals are considered to be ROS.

The redox state of the epidermal stem cells, as indicated by changes in ROS such as superoxide, could affect the developmental responsiveness (Figure 1 shows a diagram of ROS  production in mammalian cells). It has been speculated that generation of ROS can directly affect gene expression by altering chromatin configuration [28]. Thus a low cellular concentration of superoxide could directly affect DNA methylation states. This idea has merit as the activity levels of the antioxidant enzymes, superoxide dismutase (SOD), catalase, and glutathione peroxidase can change dynamically in cells. These enzymes form the first-line of defense against ROS damage. SOD converts superoxide anion to hydrogen peroxide, which is converted to water by glutathione peroxidase and catalase (Figure 2). These antioxidant enzymes keep ROS levels low in all cell types.

**Figure 3.**
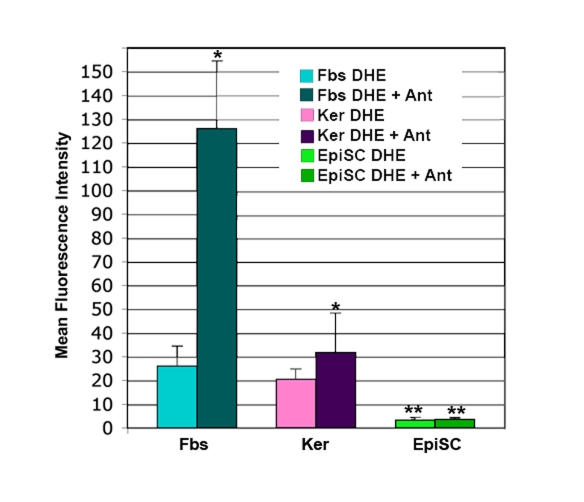
DHE staining of superoxide in skin cells. Cultures of dermal fibroblasts (Fbs), epidermal keratinocytes (Ker), and epidermal stem cells (EpiSC) were stained with dihydroethidium (DHE) in the presence or absence of the electron transport chain blocker antimycin A (Ant). Fluorescence for each cell type +/- Ant was determined by flow cytometry, then normalized by comparison to a standard cell. EpiSCs show significantly lower levels of DHE staining than the keratinocyte and fibroblast populations (p<0.01). Increase in DHE staining in the Fbs+Ant samples was significantly higher than that seen in Ker+Ant samples (p<0.05). Lack of increase in DHE staining in the EpiSC after antimycin A treatment was significant (p<0.01). Significant differences were determined by student's T-test. n=5.

Epidermal stem cells subjected to analysis of superoxide levels by dihydroethidium (DHE) show so little superoxide that it is difficult to measure (Figure 3). This is not the case with dermal fibroblasts or basal keratinocytes (Figure 3). Cells are stained with dihydroethidium (DHE). In the presence of superoxide, the DHE is reduced to ethidium, which intercalates into the DNA and fluoresces. Levels of superoxide are substantially higher in the dermal fibroblasts and basal keratinocytes than in the epidermal stem cells, as evidenced by the significant increase in fluorescence after treatment with Antimycin A (Figure 3). Antimycin A blocks electron transport at complex III, which results in a direct increase in superoxide (Figure 1). The very low level of superoxide in the epidermal stem cells is likely due to the high level of the expressed Sod1 gene previously reported [7].

In conclusion, epidermal stem cells have several characteristics that make them unique in the somatic stem cell world: They appear to resist aging. They show no age-related changes in gene expression. They maintain a developmental responsiveness to changes in their environment. They show no effects associated with increasing levels of reactive oxygen species found in aging cells by keeping levels of ROS low, perhaps by maintaining high levels of superoxide dismutase (SOD1). Exactly how these epidermal stem cells remain "young" requires further research.
